# Determinants of vegetable intake among urban socio-economically disadvantaged adolescents: a systematic review of quantitative studies

**DOI:** 10.1017/S136898002100464X

**Published:** 2022-06

**Authors:** Silvia Bel-Serrat, Antje von der Schulenburg, Amy Mullee, Celine Murrin

**Affiliations:** 1 National Nutrition Surveillance Centre, School of Public Health, Physiotherapy and Sports Science, University College Dublin, Woodview House, Belfield, Dublin 4, D04 V1W8, Ireland; 2 Department of Health and Nutritional Sciences, Institute of Technology Sligo, Sligo, Ireland

**Keywords:** Vegetable intake, Determinants, Adolescents, Socio-economically disadvantaged background

## Abstract

**Objective:**

To investigate the determinants of vegetable intake in urban socio-economically disadvantaged adolescents to inform the development of an intervention programme.

**Design:**

A narrative systematic review was carried out by searching five electronic databases from 2013 to 2020. The descriptors used for the search strategy were vegetable intake, adolescents, determinants and correlates. Inclusion criteria were including a sample of socio-economically disadvantaged adolescents aged 12–18 years, evaluation of the association between vegetable intake and determinants of intake, and conducted in urban settings of high-income countries. Thirteen studies met the inclusion criteria. Identified determinants of vegetable intake were reported according to the five levels of the socio-ecological model of health.

**Setting:**

Studies included in the review were conducted in four countries: USA (*n* 8), Australia (*n* 3), Ireland (*n* 1) and New Zealand (*n* 1).

**Participants:**

Adolescents aged 12–18 years from socio-economically disadvantaged backgrounds living in urban settings.

**Results:**

Thirty-nine determinants were identified. Nutrition knowledge was the only determinant consistently investigated in several independent samples which was not associated with vegetable intake in socio-economically disadvantaged adolescents. For the remaining potential determinants, it was not possible to examine the consistency of evidence as there were not enough studies investigating the same determinants. Most of the studies followed a cross-sectional design and were carried out in school settings.

**Conclusions:**

There is a need for further studies on the determinants of vegetable intake in this population preferably with longitudinal designs and beyond the school setting in different countries to guide the development of successful interventions.

Obesity in young populations is a serious public health challenge of the twenty-first century^(1)^. A healthy diet, which includes fruits and vegetables, is crucial in maintaining a healthy lifestyle and healthy weight^(2)^. The Health Behaviour in School-aged Children survey conducted in adolescents from Europe and Canada showed that 48 % of adolescents ate neither fruit nor vegetables daily^(3)^. This highlights that most adolescents may be far from meeting the current WHO nutrition guidelines of eating 400 g/d of fruit and vegetables^(4)^. Adolescence is a period of rapid growth and maturation. Eating behaviours are also established during this time, which will track into adulthood^(5,6)^. Hence, early intervention and promotion of healthy eating is crucial, and adolescence could represent an appropriate time for prevention efforts.

Social inequalities between those from higher and lower income also exist in terms of fruit and vegetable intake. Income has been consistently associated with intake of fruit and vegetables in young populations^(7,8)^, with significantly lower intakes of fruit and vegetables among those from poorer backgrounds^(3)^. Therefore, intervention studies aiming to promote fruit and vegetable intake in socio-economically disadvantaged adolescents should be a priority. However, it is important to explore the determinants of these behaviours in socio-economically disadvantaged adolescents in order to develop effective interventions. Although previous reviews have investigated determinants of fruit and vegetable intake in children and adolescents^(7,9)^, only the review by Di Noia and Byrd-Bredbenner^(10)^ targeted socio-economically disadvantaged children and adolescents. The review identified race/ethnicity, fruit and vegetable preferences, and maternal fruit and vegetable intake as determinants of fruit and vegetable intake in this population group. However, none of these reviews focused exclusively on vegetables or adolescents.

Vegetable intake among adolescents is lower than that of fruits, and older adolescents (15 years old) tend to have lower vegetable intake than younger adolescents (11 years old)^(3)^. Although fruit and vegetable intake is usually assessed in combination in most studies because they share certain health benefits due to their content in bioactive compounds^(11–13)^, they differ in other many aspects. They have different content of sugars, protein and fibre^(13)^ and vegetables usually need to be processed prior to their consumption. In addition, fruits and vegetables taste differently, have different textures and are consumed in different manners. While fruits are mostly sweet and are usually consumed raw as a snack, drink or a dessert, vegetables can taste bitter, often need to be cooked and are frequently consumed as part of a meal rather than as a snack^(14–16)^. In fact, taste, appearance, liking and the food environment are important determinants of vegetable intake among adolescents^(11)^. These differences between fruits and vegetables may suggest that they do not share the same determinants of intake and that intervention studies in young populations may need to target fruit and vegetable intake separately.

The ultimate purpose of this review is to inform the development of an intervention programme to promote vegetable intake in 13–15-year-old adolescent boys and girls living in socio-economically disadvantaged areas of an urban setting in a high-income country regardless of their weight status. Hence, this systematic review aimed to identify the determinants of vegetable intake that have been investigated in urban socio-economically disadvantaged adolescents. To the best of our knowledge, no systematic reviews focusing on vegetable intake in this population group have been conducted to date.

## Methods

We conducted a systematic review in accordance with the Preferred Reporting Items for Systematic Reviews and Meta-Analyses^(17)^. The systematic review was registered with the International Prospective Register of Systematic Reviews (PROSPERO) with registration ID CRD42020188213.

Given that there is a lot of variability in when adolescence occurs, we considered the different stages aligned with school systems, that is pre-school (< 6 years), primary school (6–12 years) and secondary school (12–18 years), to specify the age range for this review. Therefore, we defined adolescence as the period between 12 and 18 years.

### Search strategy

We conducted literature searches between 13 February and 5 October 2020 to identify relevant studies. Five electronic bibliographic databases were searched: PubMed, Web of Science, Cinahl, Eric and PsycINFO. The search was performed by combining key search terms for the following three categories: vegetable, population of interest (e.g. adolescents, youth), determinants and correlates (e.g. determinants, correlates, barriers, attitudes, knowledge, beliefs). No specific keywords were used for socio-economic status to retrieve as many studies as possible. The search carried out in PubMed is provided in online supplementary material, Supplemental Table S1. We also conducted manual searches of reference lists of previously published reviews and included papers to identify additional studies for consideration.

### Inclusion and exclusion criteria

Studies that met all the following criteria were included in the review: (1) sample comprised of socio-economically disadvantaged individuals (or with largest percentage of disadvantaged) described as such by the study researchers or the study was conducted in a setting described as socio-economically disadvantaged^(10)^ or comparing non-socio-economically disadvantaged *v*. socio-economically disadvantaged adolescents, (2) study participants aged between 12 and 18 years (or with a mean age between 12 and 18 years), (3) vegetable intake examined separately as an outcome, (4) association between vegetable intake and at least one hypothesised determinant of intake examined, (5) conducted in urban settings of high-income countries^(18)^, (6) English-, French-, Spanish-, Portuguese- or Catalan-language reports, (7) published in peer-reviewed journals and (8) published between 2013 and October 2020 (to avoid overlap with the review by Di Noia and Byrd-Bredbenner^(10)^ which included studies published between 2003 and August 2013. The search was limited to studies conducted in urban settings of high-income countries due to the fact that the findings of this systematic review will be used to inform the development of an intervention programme for socio-economically disadvantaged adolescents living in a large city of a European country. The languages were selected based on the authors’ knowledge of these languages.

Studies were excluded if they: (1) had a qualitative methodology or methodological aims or were meta-analyses or intervention studies, (2) were not conducted in healthy populations, (3) exclusively focused on participants with overweight and obesity and (4) exclusively focused on socio-demographic determinants such as sex, age, socio-economic position, race/ethnicity or urbanisation.

### Study selection, data extraction and data synthesis

Two reviewers (SBS and AM) independently screened titles and abstracts of 10 % of all the retrieved articles against the study selection criteria. Then, one reviewer (SBS) screened the remaining 90 % of the articles and excluded irrelevant records. Full texts were assessed when the abstract had insufficient information to make conclusions about inclusion. Again, 10 % of full-text papers that either met the eligibility criteria or had insufficient information in the abstract to determine eligibility were independently reviewed by two reviewers (SBS and AM). After discussing any disagreements, one reviewer (SBS) reviewed the full text of the remaining papers and determined the final pool of articles included in the review. Discrepancies during the screening of titles and abstracts and during the review of the full texts were discussed and agreed between the two researchers. Therefore, there was no need to involve a third researcher.

Two independent reviewers (SBS and AVDS) performed data extraction using an Excel spreadsheet to collect key data from each study. Information was extracted on first author and year of publication, study design, theoretical framework applied, study population characteristics (sample size, age, sex, race/ethnicity, setting and country), the intake assessment method applied, the study outcome, the type of analysis conducted to analyse associations, the hypothesised determinants and the presence and direction of associations with intake (considered significant when the reported *P* value was less than 0·05) for each of the determinants studied. The extracted items were drawn from prior reviews in order to allow comparisons among studies^(7,9,10)^. If associations were examined using both univariate and multivariate analyses, only multivariate analyses were reported^(9,10)^.

We conducted a narrative synthesis to summarise data on study characteristics and on the results of the included studies. The socio-ecological model of health^(19)^ was used to report on the determinants of vegetable intake identified by the included studies into five levels: personal factors, interpersonal (family- and peers-related factors), organisational (school-related factors), community-related factors and policy-related factors.

We did not conduct a quantitative synthesis, but a qualitative synthesis, due to the exploratory nature of this review. Following the reviews by Di Noia and Byrd-Bredbenner^(10)^ and McClain *et al*.^(9)^, a consistent association was defined as ‘having a relationship in the same direction over 60 % of the time as seen in at least two independent articles.’

### Study quality assessment

Two independent reviewers (SBS and AVDS) assessed the quality of each included study using an adapted version of the Newcastle-Ottawa scale for cohort and cross-sectional studies^(20,21)^. The quality assessment form included component ratings for the following criteria: (1) sample selection including representativeness of the sample, sample size, selection of the non-exposed cohort (only for cohort studies), non-respondents (only for cross-sectional studies), ascertainment of exposure and demonstration that the outcome of interest was accounted for at the start of study (only for cohort studies); (2) comparability and (3) outcome including assessment of outcome, ascertainment of outcome, length of follow-up (only for cohort studies), adequacy of follow-up cohorts (only for cohort studies) and statistical test. Sample size and statistical test were not initially included in the assessment criteria for cohort studies, but due to their relevance they were added in the tool used in this review (see online supplementary material, Supplemental Tables S2 and S3). Each criterion was assessed based on the quality assessment criteria. For each criterion, a star (+) was given if ‘yes’ was the response, whereas no star was given otherwise (i.e. an answer of ‘no’, ‘not applicable’, ‘not reported’ or ‘cannot determine’). Each star (+) was assigned a score of 1 point and a score of 0 was assigned when there was no star. For example, the criterion ‘representativeness of the sample’ within the sample selection criteria was rated as follows: (a) sample truly representative of the average in the target population (all subjects or random sampling) (1 star (+)), (b) sample somewhat representative of the average in the target population (non-random sampling) (1 star (+)), (c) sample is a selected group of users/convenience sample (0 star) or (d) no description of the derivation of the included subjects (0 star). A study-specific global score was calculated by summing up the stars across all criteria. The overall score ranged from 0 to 14 for cohort studies and from 0 to 11 for cross-sectional studies. Studies were then classified based on the final score as very good (overall score 13–14 points for cohort studies and 10–11 points for cross-sectional studies), good (overall score 10–12 points for cohort studies and 8–9 points for cross-sectional studies), satisfactory (overall score 7–9 points for cohort studies and 6–7 points for cross-sectional studies) and unsatisfactory (overall score 0–6 points for cohort studies and 0–5 points for cross-sectional studies).

## Results

### Study selection

**Fig. 1:**
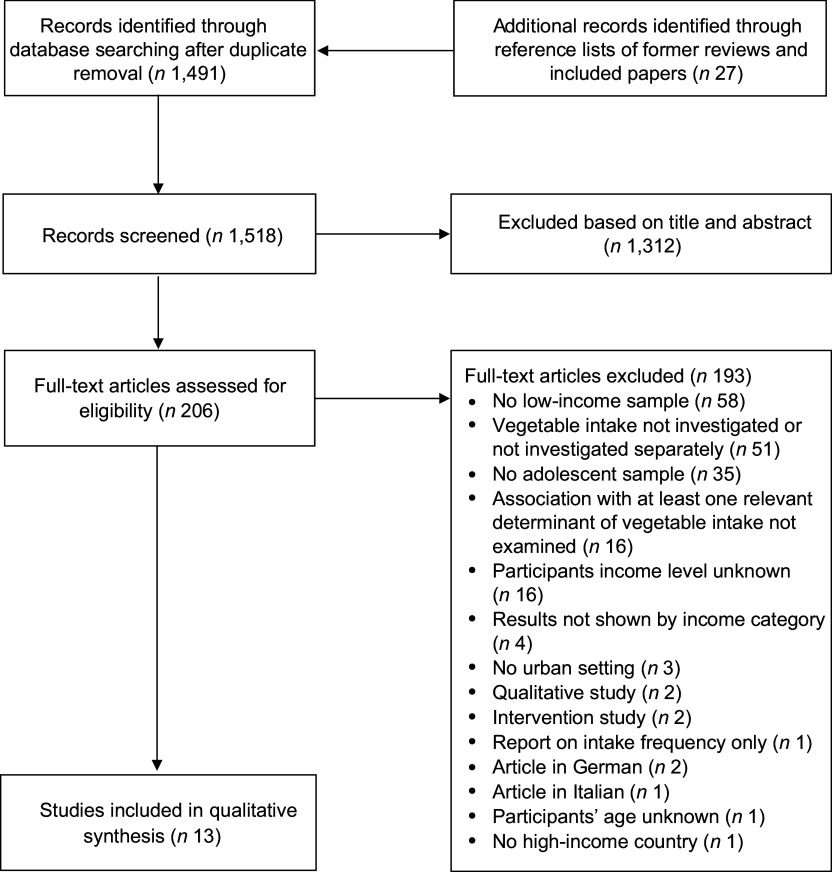
Flow chart of the study selection process

Figure 1 shows the flow chart of the study selection process. We screened 1518 articles after removal of duplicates, of which 1312 were excluded upon review of titles and abstracts. The full texts of the remaining 206 articles were reviewed against the study selection criteria, and 193 articles were further excluded. Thirteen studies matched all the inclusion criteria and were included in the present review.

### Study characteristics

Table 1 summarises the basic characteristics of the thirteen included studies, including ten cross-sectional studies^(22–31)^ and two longitudinal studies^(32,33)^ One paper reported data on both a cross-sectional and a longitudinal study^(34)^. Most of the papers were conducted in the USA (*n* 8)^(22–24,26,27,29,30,34)^, while three were carried out in Australia^(28,32,33)^, one in Ireland^(25)^ and one in New Zealand^(31)^. Sample sizes ranged between 104^(34)^ and 8500^(31)^. Five papers^(24,27,29,30,32)^ had sample sizes < 500 participants. Ten papers were conducted in the school setting: seven in high or secondary schools^(24,25,28,29,31,33,34)^, one in a middle school^(26)^, one included both primary and secondary schools^(32)^ and one study did not specify the type of school^(22)^. Other study settings included the participants’ households^(23)^, a paediatric clinic^(27)^ and neighbourhood recreation centres^(30)^.

**Table 1:** Descriptive characteristics of the studies included in the systematic review

Author, year	Design	Theoretical framework	Population characteristics (sample size, age, sex, race/ethnicity, setting, country)	Intake assessment method	Outcome measure	Analysis of associations between hypothesised determinants of intake	Hypothesised determinant(s)	Association/group with highest level of intake of vegetables
Andersen-Spruance, 2015	Cross-sectional	None specified	Sample size: 702. Age: ≤ 13 years to ≥ 16 years (7th–12th graders). Sex: boys (47·9 %) and girls (52·1 %). Race/ethnicity: African American (83·1 %). Setting: public schools. Country: USA	Questionnaire	Salad bar use (yes/no)	Multivariate	1. Healthy food preference (low/high). 2. Respondent encouragement (low/medium/high). 3. Nutrition knowledge (low/high)	1. Positive: high. 2. Positive: high *v*. low, medium *v*. low, high *v*. medium. 3. No association
Fleary, 2019	Cross-sectional	Food parenting practices content map (Vaughn, 2015)	Sample size: 1859 parent–adolescent dyads. Age: 12–17 years, 12–14 years (45·2 %), 15–17 years (45·3 %). Sex: boys (44·9 %) and girls (45·3 %). Race/ethnicity: Non-Hispanic white (57·1 %). Setting: household. Country: USA	Questionnaire	Frequency vegetable intake (times/day or week)	Univariate and multivariate	1. Supplemental food assistance	1. No association
Geers, 2017 (study 1)	Cross-sectional	Behavioural affective association model	Sample size: 1499. Age: 13–18 years (mean: 15 years, 9th–12th graders). Sex: boys (43·4 %) and girls (56·1 %). Race/ethnicity: African American (71·2 %). Setting: public high schools. Country: USA	Questionnaire	Frequency vegetable intake (times/d or week)	Multivariate	1. Positive affective associations with vegetable intake. 2. Negative affective associations with vegetable intake. 3. Cognitive beliefs on vegetable intake	1. Positive association. 2. No association. 3. No association
Geers, 2017 (study 2)	Longitudinal	Behavioural affective association model	Sample size: 104. Age: 13–18 years (mean: 15 years, 9th–12th graders). Sex: boys (49 %) and girls (51 %). Race/ethnicity: African American (90·4 %). Setting: public high schools. Country: USA	Questionnaire	Frequency vegetable intake (times/d or week)	Multivariate	1. Positive affective associations with vegetable intake. 2. Negative affective associations with vegetable intake. 3. Cognitive beliefs on vegetable intake. 4. Subjective norms on vegetable intake	1. Positive association. 2. No association. 3. No association. 4. No association
Greer, 2018	Cross-sectional	Socio-ecological model	Sample size: 327. Age: 9th–12th graders. Sex: boys (42·8 %) and girls (57·2 %). Race/ethnicity: n/a. Setting: public high schools. Country: USA	Questionnaire	Adequate intake (≥ 3 cups) *v*. non-adequate intake (< 3 cups)	Univariate	1. Having a vegetable garden at home. 2. Prior community garden/farm experience	1. Positive association. 2. No association
Kelly, 2019	Cross-sectional	None specified	Sample size: 5344. Age: <14 years (25 %), 14 years (23·8), 15 years (21 %), ≥ 16 years (30 %). Sex: boys (55·7 %) and girls (44·3 %). Race/ethnicity: n/a. Setting: post-primary schools. Country: Ireland	Questionnaire	Daily vegetables intake	Multivariate	1. School socio-economic level	1. Negative association
Marks, 2015	Longitudinal	None specified	Sample size: 243. Age 11–13 years (6th and 7th graders). Sex: boys (40·3 %) and girls (59·7 %). Race/ethnicity: 85 % born in Australia. Setting: primary and secondary schools. Country: Australia	Questionnaire	Usual intake school-day vegetables (servings/d). Usual daily frequency of vegetable consumption (servings/d)	Multivariate	1. Change of school during transition from primary to secondary school	1. No association
Roth, 2018	Cross-sectional	None specified	Sample size: 4773. Age: 7th graders. Sex: boys (50 %) and girls (50 %). Race/ethnicity: 69·5 % Latino origin. Setting: middle schools. Country: USA	FFQ	High (≥ 3 times per day) *v*. low vegetable intake	Multivariate	1. My Plate campaign knowledge. 2. Fruits and Veggies – More Matters campaign knowledge	1. No association. 2. Positive association
Saxe-Custack, 2019	Cross-sectional	None specified	Sample size: 261 caregiver–adolescent dyads. Age: mean: 12·8 years. Sex: boys (46 %) and girls (54 %). Race/ethnicity: 79·7 % African Americans. Setting: paediatric clinic. Country: USA	Questionnaire	Frequency and amount vegetable intake	Univariate	1. Food security. 2. Water safety. 3. Cost. 4. Purchasing in the neighbourhood. 5. Distance from the store. 6.Variety of good-quality vegetables	1. No association. 2. No association. 3 .No association. 4. No association. 5. No association. 6. No association
Shrewsbury, 2018	Cross-sectional	None specified	Sample size: 2538. Age: 13–14 years (8th graders). Sex: boys (47 %) and girls (53 %). Race/ethnicity: n/a. Setting: high schools. Country: Australia	Questionnaire	Daily vegetables intake (≥ 5 serves/d)	Multivariate	1. School socio-economic level	1. No association
Spruance, 2017	Cross-sectional	Socio-ecological model	Sample size: 382. Age: 7th–12th graders. Sex: boys (46 %) and girls (54 %). Race/ethnicity: 87 % African American. Setting: secondary schools. Country: USA	Questionnaire and direct observation	Salad bar use (yes/no)	Multivariate	1. Healthy food preference (low/high). 2. Nutrition knowledge (low/high). 3. Respondent encouragement (low/high). 4. Perceived parental/peer modelling (low/medium/high). 5. School wellness policy. 6. Length of school lunch (≤ 26 min, > 26 min). 7. Salad bar marketing (weak/strong). 8. Nutrition education (no/yes). 9. Other nutrition programmes (no/yes). 10. Salad bar offerings at lunch (< 5 items, ≥ 5 items). 11. Vending machines at school (no/yes). 12. Nutrition posters (no/yes). 13. Food advertisements (no/yes). 14. Attitudes of food service staff towards salad bars (negative-neutral/positive)	1. No association. 2. No association. 3. Positive association: high. 4. No association. 5. No association. 6. No association. 7. Positive association: strong. 8. No association. 9. No association. 10. No association. 11. No association. 12. No association. 13. No association. 14. No association
Stephens, 2014	Longitudinal	Socio-ecological model	Sample size: 521. Age: 12–15 years. Sex: boys (43 %) and girls (57 %). Race/ethnicity: n/a. Setting: secondary schools. Country: Australia	FFQ	Frequent vegetable intake (≥ 2 times/d)	Multivariate	1. Spending money per week. 2. Vegetables served at dinner	1. Positive association: $5–$9 spending money/week *v*. ≥ $20 spending money/week at baseline (ref). 2. Positive association: always
Trude, 2016	Cross-sectional	Social cognitive theory	Sample size: 285 caregiver–adolescent dyads. Age: 10–11 years (44·2 %), 12–14 years (55·8 %). Sex: boys (45·8 %) and girls (54·2 %). Race/ethnicity: African American (100 %). Setting: neighbourhood recreation centres. Country: USA	FFQ	Servings vegetables per day	Multivariate	1. Outcome expectancy. 2. Food knowledge. 3. Intentions. 4. Self-efficacy. 5. Youth food purchasing frequency: supermarket/corner store/convenience store/fast food. 6. Food assistance: breakfast at school/lunch at school. 7. Youth food preparation. 8. Household food purchasing frequency: supermarket/corner store/convenience store/fast food. 9. Supplemental food assistance. 10. Household food preparation score	1. No association. 2. No association. 3. Positive association. 4. Positive association. 5. Positive association: supermarket. 6. No association. 7. No association. 8. Negative association: fast food. 9. No association. 10. No association
Utter, 2016	Not reported	None specified	Sample size: 8500. Age: 9th–13th graders. Sex: boys and girls Race/ethnicity: n/a Setting: secondary schools. Country: New Zealand	Questionnaire	Frequency vegetable intake (> 3 times/d)	Multivariate	1. School garden	1. No association

All papers included mixed gender samples and ages ranged from 10 to 17 years, with four papers^(24,26,29,31)^ only reporting the school grades instead of the participants’ age. Although the targeted age range for the review was 12–18-year-adolescents, studies with adolescents < 12 years were included when the sample mean age was > 12 years or the majority of the adolescents were > 12 years^(30,32)^. Five papers were mainly conducted in African American populations^(22,27,29,30,34)^, one among Latino-origin participants^(26)^, one among non-Hispanic White participants^(23)^ and one among mainly Australian-born subjects^(32)^. Five papers did not provide any information about the ethnic origin of their sample^(24,25,28,31,33)^. Three papers^(23,27,30)^ included caregiver–adolescent dyads, whereas the remaining ten papers only included adolescents.

Six papers were based on a theoretical framework: three used the socio-ecological model^(24,29,33)^, one the social cognitive theory^(30)^, one the food parenting practices content map^(23)^ and another one the behavioural affective association model^(24)^. The remaining papers were not based on any theoretical framework or did not provide any information on the guiding conceptual framework^(22,25–28,31,32)^. In three papers, the instrument applied for measuring vegetable intake was a FFQ^(26,30,33)^. Nine papers^(22–25,27,28,31,32,34)^ assessed vegetable intake with a questionnaire and one paper combined a questionnaire with direct observation^(29)^. Multivariate analyses were carried out in ten papers^(22,25,26,28–34)^ while two papers included univariate analyses only^(24,27)^. One paper included both univariate and multivariate analyses^(23)^.

### Determinants of vegetable intake

We grouped the thirty-nine determinants of vegetables intake identified into personal factors, family- and peers-related factors, school-related factors, community-related factors and policy-related factors (Table 2). Most of the studies exclusively included samples of socio-economically disadvantaged adolescents^(22,24,26,27,29,30,32–34)^ and findings are described for the entire sample. However, for those studies that included samples of both socio-economically disadvantaged and non-socio-economically disadvantaged adolescents^(23,25,28,31)^, results are reported for one group in comparison with the other.

**Table 2:** Summary of determinants of vegetable intake in socio-economically disadvantaged adolescents

Determinant variable	Association (reference number)	No association (reference number)
Personal factors		
Cognitive beliefs		^(34)^
Encouragement towards others	Positive:^(22,29)^	
Food preparation		^(30)^
Food purchasing frequency	Positive: supermarket only^(30)^	
Intentions	Positive:^(30)^	
Negative affective associations		
Nutrition knowledge	Positive:^(26)^	^(22,26,29,30)^
Outcome expectations		^(30)^
Positive affective associations	Positive:^(34)^	
Preference	Positive:^(22)^	^(29)^
Self-efficacy	Positive:^(30)^	
Subjective norms		^(34)^
Overall adolescents weekly spending	Positive:^(33)^	
Family- and peers-related factors		
Food purchasing frequency	Negative: fast food^(30)^	
Household food security		^(27)^
Home garden	Positive:^(24)^	
Parental modelling		^(29)^
Peers modelling		^(29)^
Vegetables served with dinner (home accessibility)	Positive:^(33)^	
School-related factors		
Attitudes food service staff to salad bars		^(29)^
Availability salad bar at school lunch		^(29)^
Food advertisements		^(29)^
Length of school lunch		^(29)^
Other foods: vending machines availability		^(29)^
Receipt of school lunch subsidies		^(30)^
Salad bar marketing	Positive:^(29)^	
School change primary to secondary school		^(32)^
School garden		^(31)^
School nutrition education		^(29)^
School nutrition programmes*		^(29)^
School socio-economic level	Negative:^(25)^	^(28)^
School wellness policy		^(29)^
Community-related factors		
Community garden/farm		^(24)^
Cost		^(27)^
Stores accessibility (distance to stores)		^(27)^
Stores availability		^(27)^
Stores product quality		^(27)^
Water safety		^(27)^
Policy-related factors		
Supplemental food assistance		^(23,30)^

* Including cooking courses, farm-to-school, school garden, etc.

#### Personal factors

A total of thirteen personal factors were investigated. Nutrition knowledge was the personal factor most commonly studied in the papers included in this review. A positive significant association with vegetable intake was only found in one study^(26)^, but only for one nutrition knowledge construct, that is Fruits and Veggies – More Matters campaign knowledge, of the two knowledge constructs investigated. In this study, the nutrition knowledge construct was operationalised through the knowledge of two campaigns: the Fruits and Veggies – More Matters campaign and the MyPlate campaign. Three papers did not observe a significant association with nutrition knowledge^(22,29,30)^. Preference for healthy foods was positively associated with vegetable intake in one study^(22)^, whereas no association was found in the study by Spruance *et al*.^(29)^ Geers *et al*.^(34)^ investigated the association of several constructs within the behavioural affective association model. Positive affective associations towards eating vegetables were positively associated with vegetable intake; no association was seen for negative affective associations, cognitive beliefs and subjective norms and vegetable intake though. Trude *et al*.^(30)^ investigated psychosocial determinants of vegetable intake using constructs of the social cognitive theory. The authors reported positive associations between participants’ intention and self-efficacy for healthy eating and vegetable intake. Nevertheless, no association between outcome expectations and vegetable consumption was observed. Likewise, and focusing on food behaviour determinants, Trude *et al*.^(30)^ did not find any significant associations between food preparation and food purchasing frequency in different venues, except for purchase frequency in supermarkets which was positively associated with an increase in vegetable intake. A consistent positive association was observed in two studies^(22,29)^ between the adolescents encouraging others (peers/parents) to eat vegetables and their own vegetable intake. However, it should be noted that the results were derived from a related sample and they cannot be regarded as independent findings. Adolescents with less weekly spending of money at baseline were found to eat vegetables less frequently at follow-up than those with greater weekly spending^(33)^.

#### Family- and peers-related factors

The influence of one peer-related factor was examined. Nine family-related factors and the interaction between four of these nine family-related factors were investigated. Household meals as a determinant of vegetable intake were investigated in one study^(33)^. Always having vegetables served at dinner was associated with higher adolescents’ vegetable intake. Modelling by either parents or peers in eating vegetables was not associated with increased vegetable intake among adolescents^(33)^. Having a vegetable garden at home was positively associated with adolescents’ higher vegetable intake^(24)^. Household food purchasing frequency in fast foods was inversely associated with adolescents’ vegetable intake in one study^(30)^, whereas no association was found for frequency of purchasing in other venues such as corner stores, convenience stores and supermarkets. No association between household food security and vegetable intake in adolescents was observed in the study that investigated this association^(27)^.

#### School-related factors

At the school level thirteen constructs were examined. The socio-economic status level of the school was inversely associated with adolescents’ vegetable intake^(25)^. Adolescents attending socio-economically disadvantaged schools were less likely to eat vegetables daily than those attending schools not classified as socio-economically disadvantaged. In contrast, another study did not find a significant association between school socio-economic status and vegetable intake among adolescents^(28)^. One study^(32)^ evaluated the effect of changing schools between primary and secondary education on vegetable intake, but they did not find significant differences between those who changed school and those who did not. Spruance *et al*.^(29)^ examined a wide range of school-related factors (school wellness policy, school nutrition education, school nutrition programmes, availability of salad bar at school lunch, length of school lunch, food service staff attitudes to salad bars, food advertisements and availability of vending machines) in relation to school-based salad bar use among adolescents. No significant associations were observed between any of these factors and salad bar use among adolescents, except for salad bar marketing which was positively associated with salad bar use. Neither receipt of school lunch subsidies^(30)^ nor school garden availability^(31)^ was associated with increased vegetable intake in socio-economically disadvantaged adolescents.

#### Community- and policy-related factors

A total of six community-related factors were investigated in two papers^(24,27)^ including: prior experience with community garden/farm, availability and accessibility of stores in the neighbourhood, variety of good-quality vegetables in stores, cost of vegetables and water safety. None of these factors was significantly associated with vegetable intake among deprived adolescents.

Supplemental food assistance, the only policy-related factor investigated, was not associated with vegetable intake among adolescents in any of the two studies that examined this association^(23,30)^.

### Study quality assessment

Online supplementary material, Supplemental Tables S2 and S3 report the criterion-specific and global ratings for quality assessment for cross-sectional and longitudinal studies, respectively. Most of the included studies had a satisfactory methodological quality (*n* 7); four studies were rated as good and two studies were assessed as having an unsatisfactory methodological quality.

## Discussion

This review aimed to identify the most up-to-date determinants of vegetable intake in socio-economically disadvantaged adolescents. A total of thirty-nine determinants were investigated among the thirteen papers included in the review. Encouraging others to eat vegetables was consistently associated with higher vegetable intake; however, these conclusions were drawn from two related samples and thus cannot be considered as independent findings. Therefore, except for the construct nutrition knowledge which was consistently unrelated to vegetable intake, we failed to observe a consistent pattern of association between any of these determinants and vegetable intake in this population group, probably due to the low number of papers that met all the inclusion criteria. The following reasons could potentially explain the low number of studies included in this study: (1) limited literature available in the selected population group, that is socio-economically disadvantaged adolescents aged 12–18 years; (2) outcome of interest, that is vegetable intake only and (3) inclusion criteria applied, that is studies conducted in urban settings in high-income countries. Regardless of any these reasons, this review highlights that evidence targeting vegetable intake as an individual outcome and its determinants among socio-economically disadvantaged adolescents is scarce. Besides, there is a lack of studies involving European samples. It should be noted that we applied very specific inclusion criteria due to the ultimate purpose of this review. As these findings will inform the development of an intervention for a very specific group of adolescents regardless of their weight status, that is socio-economically disadvantaged boys and girls aged 13–15 years living in an urban setting of a high-income country, we did not investigate the role of socio-demographic and anthropometric factors on vegetable intake in this review. Furthermore, we do not believe that the publication period of the studies included in this review, that is 2013–2020, has influenced the obtained results. This review further adds to the findings reported by Di Noia and Byrd-Bredbenner in their review^(10)^ by exclusively targeting determinants of vegetable intake in socio-economically disadvantaged adolescents (12–18 years). Indeed, only four out of the eighty-five studies included in that review would have met our inclusion criteria and our results would have remained unchanged.

As already highlighted by Rasmussen *et al*.^(7)^, the existence of several studies with findings in the same direction and only few cases of contradictory findings is needed to establish epidemiological evidence for a specific association. Although we could not find consistent associations for most of the determinants identified in this review, it is still important to discuss both similar and contrasting findings among those studies that investigated the same determinants. Although methodological bias could partially explain observed discrepancies in the results, these differences may also represent true differences between populations, settings, regions, countries or time periods, among others^(7)^.

As expected, we found both similarities and differences between our findings and those from previous reviews^(7,9,10,35–38)^. However, it should be kept in mind that comparisons with previous studies are limited by the fact that these reviews (1) included several young population groups, including but not limited to adolescents, (2) did not target socio-economically disadvantaged populations exclusively, with the exception of one review^(10)^ and (3) did not focus on vegetable intake exclusively but also included consumption of fruit and fruit juices.

### Personal factors

The only construct that was investigated in at least three papers was nutrition knowledge, which refers to the ability of knowing why healthy eating is important^(8)^. There was no consistent association between nutrition knowledge and vegetable intake among socio-economically disadvantaged adolescents in this review. Contrarily, Rasmussen *et al*.^(7)^ did find a consistent positive association (in six out of eight papers) between nutrition knowledge and fruit and vegetable consumption. However, all the studies except two assessed this association for fruit and vegetables combined^(39,40)^ and they did not exclusively involve adolescent samples. Likewise, the review by McClain *et al*.^(9)^ also reported a positive association between nutrition knowledge and the consumption of fruit, fruit juice and/or vegetables in six out of nine articles. Again, only three studies carried out among school-aged children investigated the association between nutrition knowledge and vegetable intake only. In that case, two studies found a positive association^(41,42)^ and one found no association^(43)^. On the other hand, Di Noia and Byrd-Bredbenner^(10)^ did not observe a consistent association with fruit. While nutrition knowledge may play an important role in promoting healthier food intakes in specific population groups, it does not seem as relevant among socio-economically disadvantaged adolescents when fruit and vegetables are the target.

Food preference is considered as one of the strongest predictors of food choices among both young and adult populations^(8)^. This is confirmed by the consistent positive association between fruit and/or vegetable preference and intake reported in previous reviews^(7,9,10)^. Our findings did not support this association though, with food preferences only investigated in two studies showing conflicting results. While one study reported a positive association between preference for vegetables and vegetable intake, the other study did not find an association. It should be noted that Spruance *et al*.^(29)^ did find a positive significant association between high preference for healthy foods and consumption of vegetable in the unadjusted model. This association did not remain significant when other individual- and school-level factors were taken into consideration. The lack of a consistent association between vegetable preference and intake in this review together with the strong association reported by others emphasises the need to further examine this association specifically targeting socio-economically disadvantaged adolescent populations. Two studies based on a sample of 7th to 12th grade adolescents reported a positive association between the adolescents themselves encouraging other family members or peers to eat vegetables and their own intake of vegetables^(22,29)^. This association has barely been investigated.

Inconsistent results have been reported previously with other person-related constructs. While Rasmussen *et al*.^(7)^ found that self-efficacy was positively associated with increased fruit and vegetable intake, McClain *et al*.^(9)^ and Di Noia and Byrd-Bredbenner^(10)^ did not observe a consistent association between self-efficacy and vegetable intake in children and adolescents. In this review, only one study reported positive associations between either self-efficacy or intention to eat healthy and vegetable intake, while no association with outcome expectations was observed. McClain *et al*.^(9)^ also found a positive association between dietary intentions and fruit and vegetable intake, whereas no association was seen by Rasmussen *et al*.^(7)^. Only few^(7)^ or none^(9)^ of the studies included in the reviews investigated the association between dietary intentions and intake separately for vegetables though. Despite previous evidence describing an association between adolescents’ vegetable intake and awareness of the importance of regular vegetable consumption for health^(44)^, outcome expectations were not consistently associated with fruit and/or vegetable intake, either together or separately, in any of the previous reviews^(7,9,10)^. Similarly, no consistent association was observed for subjective norms and fruit and vegetable intake in the review by Rasmussen *et al*.^(7)^. Likewise, in this review, Geers *et al*.^(34)^ did not observe an association between subjective norms and vegetable intake among socio-economically disadvantaged adolescents.

Positive affective associations towards vegetables, greater weekly spending and supermarket purchasing frequency were positively associated with higher vegetable intake in the present review. These constructs were not examined in previous reviews. Based on the behavioural affective association model^(45)^, affective associations seem to have a more proximal influence on health behaviours than other variables related to social cognitive theories and are thought to mediate the links between cognitive variables and health behaviour^(34)^. Findings on adolescents’ weekly spending and supermarket purchasing frequency seem to be related to the cost and availability/variety, respectively, of vegetables. Those adolescents with overall greater weekly spending ate more vegetables, which could support the idea that vegetables are considered a costly item, mainly among socio-economically disadvantaged populations. On the other hand, vegetable availability/variety is expected to be higher in supermarkets as compared with other shopping venues such as corner stores, convenience stores or fast-food outlets, where vegetables may not be present at all and other unhealthier options are highly available at a much lower price.

### Family- and peer-related factors

The social environment has a very strong influence on adolescents’ eating behaviours through modelling, reinforcement, social support and perceived norms^(8)^. For that reason, the role of family and friends on the intake of both fruit and vegetables in young populations has been investigated extensively. However, the available literature has not shown consistent findings on how family and peer modelling influence fruit and vegetable intake. While some reviews have reported a positive association between modelling and fruit and vegetable intake^(9,36,38)^, others have failed to find a consistent association^(7,10)^. Other studies have reported a positive association between parental role modelling of vegetable intake and adolescents’ vegetable intake^(46)^. The review by Di Noia and Byrd-Bredbenner^(10)^, which also targeted socio-economically disadvantaged populations, did not observe a consistent association between peer modelling and children’s and adolescents’ fruit and vegetable intake. In the study that evaluated this construct in the present review, significant associations between vegetable intake and either parental or peer modelling were not found. The lack of association with peer modelling is somewhat surprising given the strong influence that peers and peers networks play during adolescence. McClain *et al*.^(9)^ showed that modelling was positively associated with sweetened beverage intake. This may suggest that the role of modelling may be food specific, that is, intake of certain foods by parents/peers, like vegetables, which are not considered by adolescents as ‘cool’ as other foods, may not have an impact on adolescents’ intake. Other household habits such as serving vegetables at home^(33)^ or having a home garden^(24)^ have been associated with greater vegetable intake in adolescents as described in the review by Di Noia and Byrd-Bredbenner^(10)^, albeit not consistently. However, other reviews did not find a consistent association between home availability and fruit^(10)^ and vegetable^(10,36)^ intake in children^(10)^ and adolescents^(10,36)^. On the other hand, family frequency of food purchasing at fast-food restaurants had a negative impact on adolescents’ vegetable intake. Previous qualitative data have already reported how fast-food availability either in the household or in the community has a negative impact on healthy eating and/or vegetable intake among socio-economically disadvantaged adolescents^(47–51)^.

### School-related factors

Adolescents spend a considerable amount of time at school where they eat a large proportion of their total daily energy intake^(8)^. Therefore, the school food environment unavoidably plays a huge role on the food choices and dietary behaviours of the adolescents. This is particularly relevant among underserved populations as in most cases schools represent the place where they receive their main daily meals. Based on our findings, salad bar marketing and the school socio-economic status level were the only two school-related factors that were significantly associated with the intake of vegetable intake in socio-economically disadvantaged adolescents. Among the seven school-related factors identified by Rasmussen *et al*.^(7)^, participation in school lunch programmes was associated with greater fruit and vegetable intake among children and adolescents as consistently reported by three studies. No consistent associations were observed for other factors. Similarly, none of the school factors examined by Di Noia and Byrd-Bredbenner^(10)^ showed a consistent association with intake of fruits and/or vegetables in socio-economically disadvantaged children and adolescents.

### Community-related factors

Two studies included in this review examined community-related factors and they did not observe any kind of association with vegetable intake in socio-economically disadvantaged adolescents. It should be noted that these two studies were the only two rated with unsatisfactory methodological quality. Previous reviews have not specifically investigated the role of these factors on the intake of fruit and vegetables in young populations, except for the review by Van der Horst^(38)^. Consistent with our findings, they did not find an association between fruit, juice and vegetables availability in stores and their intake by adolescent boys. Despite these findings, the physical environment in the community is known to have a large impact on the eating habits of the population, including adolescents, as it influences access to and availability of foods^(8)^. Accessibility, availability and affordability of healthy foods in the community are considered as the main barriers to a healthy diet among socio-economically disadvantaged populations^(52,53)^.

At the policy level, receipt of supplemental nutrition assistance to buy healthy foods was not associated with higher vegetable intake in adolescents^(23,30)^. Although these types of programmes have many benefits for their recipients, our findings suggest that policies targeting the individuals and their families only may not be enough to promote healthy eating and vegetable intake, specifically. Additional policies may have to be put in place, mainly those targeting the environmental level.

### Strengths and limitations

There are some limitations to this review. The presence of consistent associations could not be evaluated due to the limited number of studies included in this review as only a few studies examined the same correlates of vegetable intake. In addition, studies differed in the measurement of determinants and dietary intake, samples and analyses applied which limited comparisons among them. Most of the studies were cross-sectional. Although this design can be used to identify potential theory-based associations, we could not draw conclusions about the direction and causality of the associations. Besides, all the studies used self-reported measures which are subject to measurement error and could explain inconsistencies in the results. The majority of the studies were rated as satisfactory or good in terms of methodological quality. Although only two studies were rated as having low methodological quality, none of the studies was rated as being very good, mainly due to the lack of information about the sample size and its calculation. Absence of details does not necessarily imply that the studies were not properly designed. However, this could imply that some studies were not powered enough to detect significant associations between correlates and vegetable intake. It should also be acknowledged that most of the studies were carried out in the USA and included adolescents from different ethnic backgrounds. This may limit the generalisability of the results to other countries and ethnic groups with different socio-cultural values and socio-economic circumstances. We tried to mitigate this issue by limiting the search to studies carried out in high-income countries only. However, even among high-income countries, each country has a particular socio-economic scenario. Finally, we did our best to include all the literature relevant to our research question, but other qualifying studies may have been involuntarily omitted from the review.

Strengths of the current narrative systematic review include the focus on a vulnerable population group, that is socio-economically disadvantaged adolescents, and on vegetable intake, which has not been the focus of other reviews^(7,9,10)^. The fact that we have applied a systematic approach can also be seen as a strength. Our strategy was not limited to only studies that were published in English, but studies published in five languages were considered.

## Conclusion

This review aimed to examine the determinants of vegetable intake among socio-economically disadvantaged adolescents. A total of thirty-nine determinants were identified among the thirteen studies included in the review. Encouraging others to eat vegetables was consistently positively associated with higher vegetable intake. However, these findings should be interpreted with caution as this determinant was investigated in two related samples. Nutrition knowledge was the only construct consistently unrelated to adolescents’ vegetable intake that was investigated in several independent samples. Therefore, our results suggest that intervention studies aiming to promote vegetable intake by exclusively providing nutrition education may not be effective. Other strategies, such as involving teenagers as peer-researchers to promote vegetable intake, may be more successful. For all the remaining determinants, we could not identify any consistent associations with vegetable intake since in most cases they were only investigated by one or two studies. It should be noted that most of the factors were investigated at the individual, intra-personal and school levels. Only few were examined at the community level and only one at the policy level. These findings may reflect that a lot of the burden of eating healthily still falls on individuals and their families, who are considered as those mainly responsible for their dietary choices. Vegetable intake determinants need to be targeted across all societal levels and in combination to support socio-economically disadvantaged populations to acquire better dietary habits. Therefore, the focus needs to be shifted towards other targets such as the obesogenic environment, which is frequently observed in deprived areas. More research is warranted to investigate determinants of vegetable intake in socio-economically disadvantaged youth at different levels, preferably with longitudinal designs and involving large and representative samples. Future studies should continue targeting socio-economically disadvantaged adolescents, ideally in more settings beyond the school setting, and different countries. While these conclusions are based on limited relevant evidence available, this review still points to important research gaps that need to be filled to carry out successful public health interventions in this population group.

## Supplementary material

For supplementary material accompanying this paper visit https://doi.org/10.1017/S136898002100464X. click here to view supplementary material
