# Supplementary material for: Determinants of vegetable intake among urban socio-economically disadvantaged adolescents: a systematic review of quantitative studies
Source: Public Health Nutr. 2021 Nov 24;25(6):1447–60. doi: 10.1017/S136898002100464X (PMC9991624; doi:10.1017/S136898002100464X)
Supplement: Supplementary file 1 [file S136898002100464Xsup.zip › S136898002100464Xsup002.docx]

**Table S1.** Search strategy and search terms used in PubMed.

| #1 Vegetable | vegetable* [MeSH] OR vegetable*[Title/Abstract] |
| --- | --- |
| #2 Population | adolescen* [MeSH] OR adolescen*[Title/Abstract] OR teen*[Title/Abstract] OR youth*[Title/Abstract] |
| #3 Determinants and correlates | Determinant*[Title/Abstract] OR correlate*[Title/Abstract] OR belief*[Title/Abstract] OR attitude*[Title/Abstract] OR knowledge[Title/Abstract] OR perception*[Title/Abstract] OR view*[Title/Abstract] OR intention*[Title/Abstract] OR facilitator*[Title/Abstract] OR barrier*[Title/Abstract] |
| #4 = #1 AND #2 AND #3 | ((vegetable* [MeSH] OR vegetable*[Title/Abstract]) AND (adolescen* [MeSH] OR adolescen*[Title/Abstract] OR teen*[Title/Abstract] OR youth*[Title/Abstract])) AND (Determinant*[Title/Abstract] OR correlate*[Title/Abstract] OR belief*[Title/Abstract] OR attitude*[Title/Abstract] OR knowledge[Title/Abstract] OR perception*[Title/Abstract] OR view*[Title/Abstract] OR intention*[Title/Abstract] OR facilitator*[Title/Abstract] OR barrier*[Title/Abstract]) |
| #5 | Limit #4 to humans and English, Spanish, French, Portuguese and Catalan |

**Table S2.** Study quality assessment using the National Institutes of Health’s Quality Assessment Tool for observational cross-sectional studies.

| **Author, publication year** | **Study design** | **Selection** | | | | **Comparability*** | **Outcome** | | | **Total score** |
| --- | --- | --- | --- | --- | --- | --- | --- | --- | --- | --- |
|  |  | **Representativeness sample** | **Sample size** | **Non-respondents** | **Ascertainment of exposure*** | **Based on design and analysis** | **Assessment outcome** | **Ascertainment outcome*** | **Statistical test** |  |
| **Andersen-Spruance, 2015** | Cross-sectional | + |  | + | + | ++ | + |  | + | 7 |
| **Fleary, 2019** | Cross-sectional | + |  | + | + |  | + | + | + | 6 |
| **Geers, 2017 (study 1)** | Cross-sectional |  |  |  | + | ++ | + | + | + | 6 |
| **Greer, 2018** | Cross-sectional | + |  |  |  |  | + | + |  | 3 |
| **Kelly, 2019** | Cross-sectional | + | + | + |  | ++ | + | + | + | 8 |
| **Roth, 2018** | Cross-sectional |  |  | + |  | ++ | + | + | + | 6 |
| **Saxe-Custack, 2019** | Cross-sectional |  |  |  | + |  | + | + |  | 3 |
| **Shrewsbury, 2018** | Cross-sectional | + | + | + |  | ++ | + | + | + | 8 |
| **Spruance, 2017** | Cross-sectional | + |  | + | + | ++ | + | + | + | 8 |
| **Trude, 2016** | Cross-sectional |  |  |  | + | ++ | + | + | + | 6 |
| **Utter, 2016** | Not reported | + |  | + |  | ++ | + |  | + | 6 |

For each criterion, a star (+) is given if “yes” is the response, whereas no star is given otherwise (i.e., an answer of “no,” “not applicable,” “not reported,” or “cannot determine”). Each star (+) is assigned a score of 1 point, a score of 0 is assigned when there is no star. The total score is calculated by summing up the stars across all criteria. *Newcastle-Ottawa scale adapted for cohort and cross-sectional studies, two stars (++), one star (+) or no stars can be assigned to this criterion.

The following aspects were evaluated: a) selection: sample was representative of the target population, sample size was justified and satisfactory including sample size calculation, comparability between respondents and non-respondents characteristics was established and response rate was satisfactory (>60%), and assessment of validity and reliability of methods used to measure exposure; b) comparability: subjects in different outcome groups were comparable, based on the study design or analysis, and confounding factors were controlled; and c) outcome: method used to assess outcome, validity and reliability of methods used to measure outcome, and appropriateness and reporting of the statistical test used to analyse the data.

Cross-sectional studies rating (total score 11 stars):

Very good studies: 10-11 points

Good studies: 8-9 points

Satisfactory studies: 6-7 points

Unsatisfactory studies: 0 to 5 points

**Table S3.** Study quality assessment using the National Institutes of Health’s Quality Assessment Tool for cohort studies.

| **Author, publication year** | **Study design** | **Selection** | | | | | **Comparability*** | **Outcome** | | | | | **Total score** |
| --- | --- | --- | --- | --- | --- | --- | --- | --- | --- | --- | --- | --- | --- |
|  |  | **Representativeness sample** | **Sample size** | **Selection non-exposed cohort** | **Ascertainment exposure*** | **Outcome of interest accounted for/not present at start of study** | **Based on design and analysis** | **Assessment outcome** | **Ascertainment outcome*** | **Enough length follow-up?** | **Follow-up adequacy** | **Statistical test** |  |
| **Geers, 2017 (study 2)** | Longitudinal |  |  | + | + |  | ++ | + | + |  |  | + | 7 |
| **Marks, 2015** | Longitudinal | + | + | + | n/a | + | ++ | + | + |  | + | + | 10 |
| **Stephens, 2014** | Longitudinal |  |  | + | + | + | ++ | + | + | + |  | + | 9 |

n/a, not applicable.

For each criterion, a star (+) is given if “yes” is the response, whereas no star is given otherwise (i.e., an answer of “no,” “not applicable,” “not reported,” or “cannot determine”). Each star (+) is assigned a score of 1 point, a score of 0 is assigned when there is no star. The total score is calculated by summing up the stars across all criteria. *According to Effective Public Health Practice Project Quality Assessment Tool for Observational Cohort and Cross-Sectional Studies, two stars (++), one star (+) or no stars can be assigned to this criterion.

The following aspects were evaluated: a) selection: sample was representative of the target population, sample size was justified and satisfactory including sample size calculation, non-exposed cohort was drawn from the same community as the exposed cohort, assessment of validity and reliability of methods used to measure exposure, and outcome of interest was accounted for or not present at start of study; b) comparability: subjects in different outcome groups were comparable, based on the study design or analysis, and confounding factors were controlled; and c) outcome: method used to assess outcome, assessment of validity and reliability of methods used to measure outcome, enough length of follow-up (>1 year), subjects lost to follow-up (complete follow-up or <20% attrition rate, and clear description, and appropriateness and reporting of the statistical test used to analyse the data.

Cohort/longitudinal studies rating (total score 14 stars):

Very good studies: 13-14 points

Good studies: 10-12 points

Satisfactory studies: 7-9 points

Unsatisfactory studies: 0 to 6 points
